# T-Cell Lymphoproliferative Disorders Following Allogeneic Bone Marrow Transplant: A Report of Two Cases and a Literature Review

**DOI:** 10.7759/cureus.59901

**Published:** 2024-05-08

**Authors:** Nicholas Prabhakar, Harrah Chiang, Irma Munoz Verdugo, Ari Hakimian, Shams Bufalino, Jacob Bitran

**Affiliations:** 1 Internal Medicine, Advocate Lutheran General Hospital, Park Ridge, USA; 2 Internal Medicine, Advocate Christ Medical Center, Oak Lawn, USA; 3 Hematology and Oncology, Advocate Lutheran General Hospital, Park Ridge, USA

**Keywords:** t-cell post-transplantation lymphoproliferative disorders, post bone marrow transplant complications, allogenic bone marrow transplant, allogeneic stem cell transplant, t-cell large granular lymphocyte leukemia

## Abstract

Post-transplantation lymphoproliferative disorders (PTLD) are a commonly occurring condition following solid organ transplantation (SOT) and, rarely, hematopoietic stem cell transplantation (HSCT). As the name suggests, a PTLD is a condition where there is a clonal proliferation of lymphoid cells that occurs as a complication after transplantation. Though the clonal origin cell is primarily associated with the B-cell lineage, there are existing cases in the literature describing PTLD from the T-cell lineage. Large granulocytic leukemia (LGL) is one rare T-cell lineage subtype that typically progresses with a passive clinical course and is discovered with leukocytosis and peripheral blood smears demonstrating large granules in lymphocytes.

In this study, we describe two patients initially diagnosed with acute myeloid leukemia (AML) who were both found to have T-cell PTLD after undergoing allogeneic hematopoietic stem cell transplant. One was found with a clonal expansion of T-cells on flow cytometry and the other with LGL on peripheral blood and flow cytometry. This discovery was made at 16 and 20 months after their transplant respectively. Distinguishing factors for these two patients are demonstrated by the derivation of lymphoproliferative disorder from graft vs. host disease (GVHD) or viral etiology, which is significant as both of which have been shown to be associated with PTLD. Epstein-Barr virus (EBV) and cytomegalovirus (CMV) positivity have been shown to be associated with PTLD, and both our patients were EBV-negative but had harbored prior CMV infections. Additionally, they had a benign course with no development of cytopenias or symptoms since the time of diagnosis.

These two cases add to the growing literature that is working to better characterize the rare development of LGL and, in general, T-cell PTLD following allogeneic bone marrow transplantation.

## Introduction

Post-transplantation lymphoproliferative disorders (PTLD) encompass a heterogeneous group of diseases that can occur following the transplantation of solid organs (SOT) or allogeneic hematopoietic stem cells (HSCT). As the name suggests, PTLD involves a clonal proliferation of lymphoid cells of B, T, or NK cell origin that can present with a wide phenotypic variety. The overall incidence of PTLD has been reported as occurring in 1-4% of patients after HSCT, and the risk of PTLD is highest in the first year after transplantation with the incidence declining thereafter [1,2]. The clonal population primarily arises from the B-cell lineage and is also often associated with latent Epstein-Barr virus (EBV) infection in 60-80% of patients [3,4]. However, PTLD can also rarely derive from T or natural killer (NK) cells (2-15% of all PTLD), which includes a subtype known as large granular lymphocyte leukemia (LGL). LGL is a rare chronic lymphoproliferative condition of T (CD3/CD8+) or NK cells (CD3-) that makes up 2-5% of all chronic lymphoproliferative diseases with an incidence of 0.2-0.72 per million persons per year [5,6]. Lymphocytes in LGL are larger and contain characteristic cytoplasmic azurophilic granules visible on microscopy [5]. Diagnosis is typically made when patients are found to have a persistent increase in peripheral blood LGL (typically between 2 to 20x10^9/L) without a clearly identifiable cause [7]. LGL subtypes include chronic T-cell leukemia, chronic NK cell lymphocytosis, and aggressive NK cell LGL leukemia. Over the last 20 years, there have been increasing reports of LGL occurring after HSCT. Most series detailing this phenomenon describe patients with benign clinical courses where LGL was identified after screening for lymphocytosis, but the most common complication of LGL when symptomatic is related to resultant cytopenias [8]. STAT3 mutations have also been shown to have diagnostic value in differentiating T-cell LGL from other T-cell neoplasms and reactive conditions while the expression of STAT5 is related to a more aggressive clinical presentation [9,10]. In addition, it has been reported that there is a relationship between LGL leukemia and autoimmune diseases. In LGL and other T-cell PTLD, there are well-documented connections to various viral infections, including the human T-lymphotropic virus 1 (HTLV1), hepatitis C, Epstein-Barr virus (EBV), and cytomegalovirus (CMV) [5,11]. Treatment is typically tailored with immunomodulators targeting molecular pathways related to the disease [5]. In this study, we report two cases of T-PTLD after HSCT in EBV-negative, CMV infection history positive, asymptomatic patients. These cases add to the growing literature that is working to better characterize PTLD of T cell origin including LGL. 

## Case presentation

The first case was a 71-year-old female with a past medical history of hypertension who presented to her primary care physician with a 10-day course of severe sore throat, fatigue, and low-grade fever. She received antibiotics, but after developing gross hematuria and bruising on her thighs, the patient was prompted to go to the hospital. Her laboratory findings on admission revealed leukocytosis of 23 K/mcL with 22% blasts, absolute neutrophil count (ANC) of 10.1 K/mcL, and platelets of 27 K/mcL. Bone marrow biopsy (BMB) was performed that revealed acute monoblastic leukemia, which is a subtype of acute myeloblastic leukemia (AML) with next-generation sequencing (NGS) identification of the specific genetic mutation of FLT3-TKD. The patient was started on induction chemotherapy with cytarabine 200 mg/m^2 ^continuous infusion over 24 h for 7 days, plus daunorubicin 60 mg/m^2^ slow intravenous push for 3 days, with gemtuzumab ozogamicin 4.5 mg for 3 doses. Following treatment, a repeat BMB showed cellular bone marrow with myeloid predominance without any morphological evidence of the previously diagnosed AML. Consolidation therapy was initiated with high-dose cytarabine 3,000 mg/m^2^ every 12 hours for a total of 6 doses while undergoing pre-transplant testing. The patient underwent an allogeneic matched unrelated donor (allo MUD) hematopoietic stem cell transplant five months after the initial diagnosis of AML. On allo MUD day +24, she tested positive for cytomegalovirus (CMV) and was treated. The patient proceeded with an uneventful course until Day +134 when she complained of fever and bilateral numbness in her hands and feet. An acute rash on her feet developed and was consistent with stage II acute graft vs. host disease (GVHD) of the skin, with 36% involvement of body surface area. Her symptoms improved with a course of steroids. She was discontinued on her immunosuppression with tacrolimus on Day +175. On day +226, in the setting of persistent nausea, vomiting, and diarrhea that brought the patient to the hospital, there was concern for further GVHD. An upper endoscopy was performed; however, it yielded negative results and symptoms resolved with a short course of steroids. On day +542, a repeated BMB reported chimerisms of CD3+ cells, which were 100% of donor origin. Likewise, the CD33+ cells were also 100% donor. It was slightly hypercellular bone marrow (30-40% cellular) with trilineage hematopoiesis and mild patchy 1+ fibrosis. Flow cytometry performed on day +625 demonstrated an increase in CD56/57+ T-cells and an inverted ratio of CD4+ and CD8+ cells consistent with a new low-grade T-cell/NK lymphoproliferative disorder with biclonal gammopathy. The patient was treated for her disseminated zoster, but after her hospital stay, she developed a rash on her feet, which was biopsied and found to be skin GVHD. Aside from her complicated course with the viral infection, the patient’s complete cell count (CBC) remained stable throughout her hospital stay.

**Figure 1:**
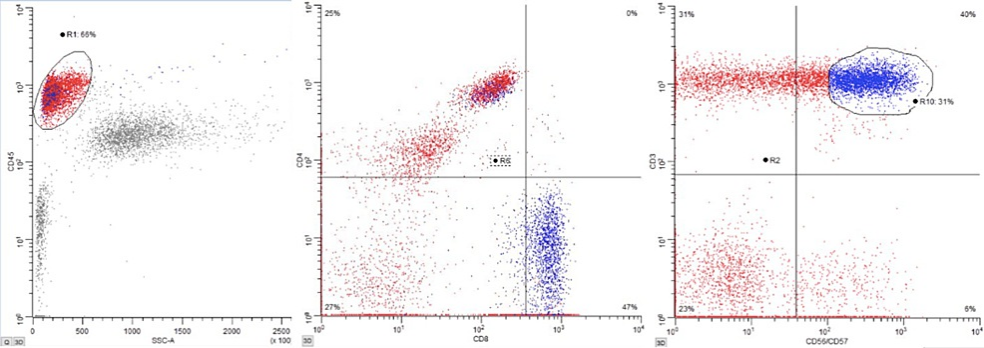
Flow cytometry showing increased CD56/57 positive T-cells with CD8 restriction

**Figure 2:**
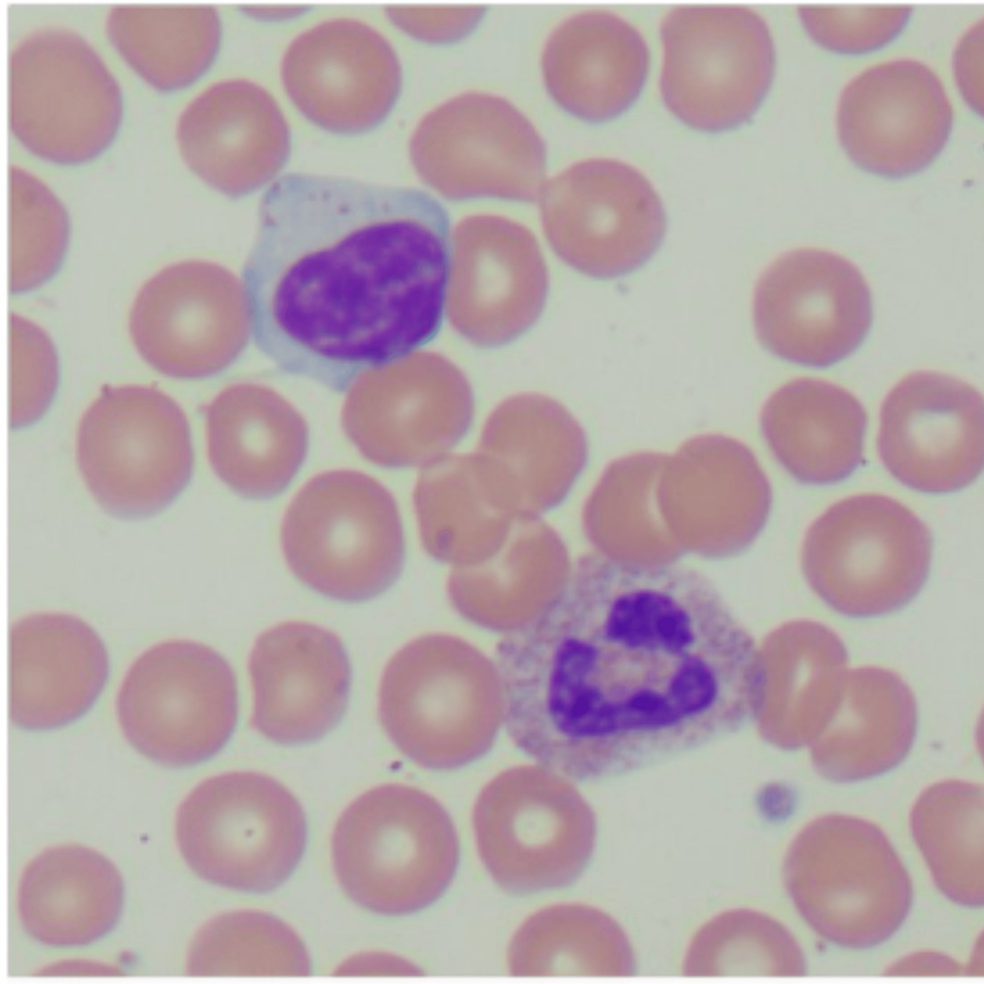
Characteristic azurophilic granules visualized in lymphocytes found on peripheral blood smear with hematoxylin and eosin staining, 100x magnification

The second patient was a 54-year-old female with a past medical history of hypertension, hyperlipidemia, morbid obesity, diabetes mellitus, sarcoidosis, polymyalgia rheumatica, and cervical and endometrial dysplasia status post total laparoscopic hysterectomy and bilateral salpingo-oophorectomy. The patient was diagnosed with acute myelogenous leukemia (AML) three years prior (cytogenetic testing was normal, FTL3 positivity with low allelic burden (<0.5) and NPM-1 positivity), and initially for induction therapy, she was treated with cytarabine 200 mg/m^2^ continuous infusion over 24 h for 7 days, plus daunorubicin 60 mg/m^2^ slow intravenous push for 3 days, and midostaurin 50 mg twice daily for 8 days; followed by high-dose cytarabine 2,000 mg/m^2^ and midostaurin 25mg twice daily for consolidation therapy. The patient was then on midostaurin at that same dose for maintenance therapy, and she was eventually found to have a relapse of AML with a new t(1;7) rearrangement after eight months. She was then admitted for re-induction salvage chemotherapy, then was started on azacitidine + venetoclax maintenance, with day 28 bone marrow showing no leukemic cells with empty bone marrow. A month after this, the patient underwent an allogeneic haploidentical stem cell transplant. The transplant hospital course was complicated by Klebsiella bacteremia, Escherichia coli urinary tract infection, and Enterococcus faecalis central venous catheter line infection. Day 100+ bone marrow biopsy was hypocellular, with mildly increased blasts (3.6% by flow cytometry), with no evidence of leukemia. At that time, immunosuppression with tacrolimus was discontinued. Prior to transplantation, the patient was noted to be seropositive for CMV and herpes simplex virus (HSV) I and II and had mild reactivation of CMV shortly after transplantation hospitalization that was effectively treated. The course remained unremarkable until 1/2024 when it was noted that the patient had persistent lymphocytosis (present for >6 months) on her CBC, with a flow cytometry that showed an increase in CD56/CD57+ T-cells with CD8 expression (Figure 1), consistent with large granular lymphocytes (65% of all lymphoid cells), supportive of a diagnosis of T-cell/NK cell large granulocytic leukemia. No cytopenias were noted at that time. Peripheral smear showed the characteristic absolute lymphocytosis with characteristic granules found in LGL (Figure 2). At the time of discovery, the patient was asymptomatic with stable vitals and has remained so through her clinical course. There have been no other changes to her cell counts, with no cytopenias or other symptoms noted in follow-up visits.

## Discussion

Due to the rarity of PTLD with T-cell lineage especially after HSCT (compared to SOT), information regarding pathogenesis, risk factors, and therapy is primarily derived from case reports and series. Risk factors that have been identified thus far for developing PTLD after HSCT include T-cell depletion via methods that selectively targeted T-cells, anti-thymocyte globulin (ATG) or anti-CD3 monoclonal antibody, chronic graft vs host disease requiring prolonged immunosuppression and unrelated or HLA mismatched grafts [1,12]. There also may be an association with early immune reconstitution and PTLD after HSCT [7]. EBV is another large risk factor primarily with PTLD of B-cell lineage, with the suspected mechanism behind this believed to be related to the delay in immune reconstitution of anti-EBV cytotoxic T-lymphocytes after myeloablation and homing of donor stem cells; this allows the EBV-positive B-cell population to proliferate and form a PTLD line [3,4]. T-PTLD is not as commonly associated with EBV positivity unlike PTLD of B-cell origin, and this was reflected in our patient cases where both patients were EBV negative [4]. Awada et al. explored many possible mechanisms of T-cell clonal expansion post-HSCT to explain T-PTLD; this included viral infections and latent viral infection reactivation, graft allo-antigenic stimulation, or reactivation of an existing autoimmune process [13]. Tiede et al. examined 163 cases of T-PTLD after both SOT and HSCT. They found that HSCT is a predisposing risk factor for the development of early-onset T-PTLD, although T-PTLD overall occurs most often in SOT patients [4]. The hypothesized reason for this development may be due to defective donor stem cells. Late-onset PTLD is less common in HSCT due to immunosuppressants and improved immunocompetence. The current recommended treatment in general for PTLD involves the reduction of immunosuppression, rituximab, and donor lymphocyte infusion [3,14]. Although LGL is typically treated with immunomodulatory medication as mentioned previously.

LGL after HSCT was first described in the literature primarily by case reports and small case series in the early 2000’s [15,16]. Since then, research has steadily increased, with multiple institutions performing retrospective and cross-sectional studies describing their experience with T-cell LGL after HSCT. This has helped elucidate potential clinical features and predisposing factors. In general, most patients in these studies with LGL were asymptomatic, and the diagnosis had no negative impact on their overall survival. While there was no universal consensus among these studies regarding risk factors and associations, multiple studies found strong links to prior CMV infection and GVHD, with one describing a connection to tyrosine kinase inhibitors [1,7,17,18]. Nann-Rutti et al. found T-LGL expansions in 7% of their patients post-HSCT, with 36% of that subset showing clonality. Their subset showed an association with CMV and all were asymptomatic with benign clinical courses related to the LGL [18]. They speculated that such expansions may instead be the product of chronic stimulation triggered by CMV expansion rather than a malignant transformation. Another interesting study found an incidence of large granular lymphocytosis in 14.5% of their post-HSCT patients over 3.5 years. In their patient subset, they also found CMV to be highly associated and found that most were asymptomatic despite the cytopenia [11]. Multiple retrospective studies have also found chronic GVHD association in addition to prior studies, with a lower non-relapse mortality in that population [16,19]. Additionally, there have been multiple groups reporting improved survival in their LGL-positive groups compared to their cohorts [1, 19]. Messmer et al. took a different angle in that they performed a retrospective study with 150 patients found to have unexplained cytopenia following allogeneic bone marrow transplant (alloBMT), and investigated these patients for LGL [8]. They found 75 of these patients to be LGL-positive with 22% showing cell receptor clonality. Compared to the LGL-negative alloBMT patients, there was no difference in a significant demographic or transplant, however, they did note that LGL-positive patients were more likely to have a history of CMV viremia, no relation to GVHD, and no difference in overall survival compared to the LGL-negative counterparts [8]. Interestingly, one of our patients who had T-cell lymphoproliferation had a history of CMV and GVHD while the other with LGL only had prior CMV, and had multiple bacteremias during their transplant course. LGL is also associated with autoimmune diseases, which is present in our second case of a patient with LGL.

## Conclusions

T-cell PTLD is a rare phenomenon that can occur following bone marrow transplantation. We aimed to add to the expanding literature exploring and characterizing T-PTLD by describing two patients with T-cell PTLD after HSCT, including one case of LGL. In regards to the common risk factors predisposing to PTLD after a transplant, both patients were EBV-negative, both had a prior CMV infection, and one patient had a history of GVHD. It is worth noting that neither of the patients developed secondary cytopenias after developing these lymphoproliferative disorders and have continued to remain asymptomatic with a benign disease course. Future directions for research involve larger-scale studies examining transplant populations found to have T-cell PTLD in order to better elucidate both risk factors and the clinical course in these patients. Further clarification of the development and disease course will be helpful for providers whose patients are undergoing HSCT and for their management after transplantation.

## References

[1] Curtis RE, Travis LB, Rowlings PA (19991). Risk of lymphoproliferative disorders after bone marrow transplantation: a multi-institutional study. Blood.

[2] Dierickx D, Habermann TM (2018). Post-transplantation lymphoproliferative disorders in adults. N Engl J Med.

[3] Uhlin M, Wikell H, Sundin M (2014). Risk factors for Epstein-Barr virus-related post-transplant lymphoproliferative disease after allogeneic hematopoietic stem cell transplantation. Haematologica.

[4] Tiede C, Maecker-Kolhoff B, Klein C, Kreipe H, Hussein K (2013). Risk factors and prognosis in T-cell posttransplantation lymphoproliferative diseases. Reevaluation of 163 cases. Transplantation.

[5] Rahul E, Ningombam A, Acharya S, Tanwar P, Ranjan A, Chopra A (2022). Large granular lymphocytic leukemia: a brief review. Am J Blood Res.

[6] Moignet A, Lamy T (2018). Latest advances in the diagnosis and treatment of large granular lymphocytic leukemia. Am Soc Clin Oncol Educ Book.

[7] Lamy T, Loughran TP Jr (2011). How I treat LGL leukemia. Blood.

[8] Messmer M, Wake L, Tsai HL, Jones RJ, Varadhan R, Wagner-Johnston N (2021). Large granular lymphocytosis with cytopenias after allogeneic blood or marrow transplantation: clinical characteristics and response to immunosuppressive therapy. Transplant Cell Ther.

[9] Koskela HL, Eldfors S, Ellonen P (2012). Somatic STAT3 mutations in large granular lymphocytic leukemia. N Engl J Med.

[10] Muñoz-Ballester J, Chen-Liang TH, Hurtado AM (2016). Persistent cytotoxic T lymphocyte expansions after allogeneic haematopoietic stem cell transplantation: kinetics, clinical impact and absence of STAT3 mutations. Br J Haematol.

[11] Poch Martell M, Hamad N, Shin E (2017). Distinctive clinical characteristics and favorable outcomes in patients with large granular lymphocytosis after allo-HCT: 12-year follow-up data. Eur J Haematol.

[12] Landgren O, Gilbert ES, Rizzo JD (2009). Risk factors for lymphoproliferative disorders after allogeneic hematopoietic cell transplantation. Blood.

[13] Awada H, Mahfouz RZ, Durrani J (2020). Large granular lymphocytic leukaemia after solid organ and haematopoietic stem cell transplantation. Br J Haematol.

[14] Styczynski J, Gil L, Tridello G (2013). Response to rituximab-based therapy and risk factor analysis in Epstein Barr Virus-related lymphoproliferative disorder after hematopoietic stem cell transplant in children and adults: a study from the Infectious Diseases Working Party of the European Group for Blood and Marrow Transplantation. Clin Infect Dis.

[15] Au WY, Lam CC, Lie AK (2003). T-cell large granular lymphocyte leukemia of donor origin after allogeneic bone marrow transplantation. Am J Clin Pathol.

[16] Chang H, Kamel-Reid S, Hussain N (2005). T-cell large granular lymphocytic leukemia of donor origin occurring after allogeneic bone marrow transplantation for B-cell lymphoproliferative disorders. Am J Clin Pathol.

[17] Kim DH, Kamel-Reid S, Chang H (2009). Natural killer or natural killer/T cell lineage large granular lymphocytosis associated with dasatinib therapy for Philadelphia chromosome positive leukemia. Haematologica.

[18] Nann-Rütti S, Tzankov A, Cantoni N (2012). Large granular lymphocyte expansion after allogeneic hematopoietic stem cell transplant is associated with a cytomegalovirus reactivation and shows an indolent outcome. Biol Blood Marrow Transplant.

[19] Kim D, Al-Dawsari G, Chang H (2013). Large granular lymphocytosis and its impact on long-term clinical outcomes following allo-SCT. Bone Marrow Transplant.

